# Vaccine immunity in patients with 22q11.2 microdeletion syndrome

**DOI:** 10.1111/pai.70043

**Published:** 2025-02-10

**Authors:** Leila Farpour, Renato Gualtieri, Tereza Kotalova, Barbara Lemaître, Julie Ducreux, Isabelle Arm‐Vernez, Stephan Eliez, Geraldine Blanchard‐Rohner

**Affiliations:** ^1^ Geneva University Hospitals and Faculty of Medicine Geneva Switzerland; ^2^ Platform for Pediatric Clinical Research, Department of Pediatrics, Gynecology and Obstetrics Geneva University Hospitals and University of Geneva Geneva Switzerland; ^3^ Pole Autism Foundation Geneva Switzerland; ^4^ Vaccinology Laboratory Geneva University Hospitals and University of Geneva Geneva Switzerland; ^5^ Flow Cytometry Lab, Diagnostic Department, Service of Clinical Pathology Geneva University Hospitals Geneva Switzerland; ^6^ Virology Laboratory Geneva University Hospitals and University of Geneva Geneva Switzerland; ^7^ Department of Psychiatry, Faculty of Medicine University of Geneva Geneva Switzerland; ^8^ Unit of Immunology, Vaccinology, and Rheumatology, Division of General Pediatrics, Department of Pediatrics, Gynecology and Obstetrics Geneva University Hospitals and University of Geneva Geneva Switzerland

**Keywords:** 22q11.2 microdeletion syndrome, 31 vaccination, immunology workup, vaccine immunity

## Abstract

**Background:**

Patients with microdeletion 22q11.2 syndrome (MDS) exhibit immunological defects, characterized by abnormalities in the development of the thymus, which plays a crucial role in T‐cell maturation and immune response. As a result, these patients may have impaired adaptive immunity, with decreased responses to vaccination.

**Methods:**

This was a prospective observational study. Vaccine serology (tetanus, diphtheria, *Haemophilus influenzae* type b for children <5 years, measles, varicella, hepatitis A and B, and SARS‐CoV‐2) and immune parameters were assessed in MDS patients aged between 1 and 25 years followed in Geneva between February 2022 and April 2023.

**Results:**

41 MDS patients were included. The median age was 13 years old. Most of them reported recurrent otitis and bronchitis up to 10 years, and a mild COVID‐19 disease in the past. Immunological work‐up indicated normal immunoglobulin levels and lymphocyte counts for the majority. Most patients were well vaccinated for tetanus, diphtheria, *Haemophilus influenzae* type b and measles, but only half were fully vaccinated for hepatitis B, and SARS‐CoV‐2 and only a quarter for hepatitis A. 70% of the patients had received 3 doses of pneumococcal conjugate vaccine in infancy but only a minority an additional dose. While most of them were seroprotected against tetanus, diphtheria, and Hib, a substantial number lacked seroprotection against varicella, measles, hepatitis B, and pneumococcus.

**Conclusion:**

This study suggests that regular assessment of antibody levels for measles, hepatitis B, varicella, and pneumococcus, regardless of vaccination status should be encouraged in MDS patients, with reimmunization according to vaccine serology, to enhance vaccine immunity.

**Key message**

The findings reveal a significant decrease in vaccine immunity in patients with 22q11.2 microdeletion syndrome, particularly against varicella, measles, hepatitis B, and pneumococcus. These insights are crucial for guiding clinicians toward more effective vaccination strategies for this population, suggesting that additional vaccine doses may be necessary for these patients, even with normal immunological workups.

## 1 INTRODUCTION

Microdeletion 22q11.2 syndrome (MDS), also known as DiGeorge syndrome, is one of the most common polymalformative genetic disorders with a prevalence of 1/2000 live births.^1^ This syndrome includes, with variable frequency, facial dysmorphism, heart disease, hypoparathyroidism responsible for hypocalcemia, and hypoplasia of the thymus that can lead to immunodeficiency.^2^

These children may also develop psychiatric problems such as anxiety disorders, mood disorders, attention deficit with or without hyperactivity (ADHD).^3^ It is therefore recommended that these children have a child psychiatric follow‐up.

Most patients with MDS have a moderate to a mild immunodeficiency and/or autoimmune pathologies. Indeed, due to the hypoplasia of the thymus, these patients have a diminished T‐cell production and function.^4^, ^5^ In <1% of cases, the immunodeficiency is major. Previous research on the immune response to vaccination in this population has reported lower antibody responses but good tolerance to live vaccines even in patients with abnormal immunology workup (see Table S4).^6^, ^7^, ^8^, ^9^, ^10^, ^11^, ^12^, ^13^, ^14^, ^15^, ^16^ Currently, vaccines are recommended as follows: non‐live vaccines should be administered according to the standard vaccination schedule. However, an additional dose of the pneumococcal vaccine is recommended in France and Belgium (see Table S4).^17^, ^18^ It should be noted that the vaccines especially recommended for immunocompromised persons include pneumococcal, yearly influenza, Hib, meningococcal, and hepatitis B vaccines.^19^ Concerning live vaccines, according to the American Academy of Pediatrics Red Book guidelines, all live bacterial and viral vaccines are contraindicated in cases of complete and partial DiGeorge syndrome. However, they mention that children with a CD3 count ≥500 cells/mm^3^, CD8 counts ≥200 cells/mm^3^, and normal mitogen responses, can be considered for MMR and varicella vaccines (but not MMRV).^20^ In contrast, a recent expert consensus suggests administering MMR and varicella vaccines at 12 months of age, if the following criteria are met: a CD4 count ≥400 cells/mm^3^, a CD8 count ≥200 cells/mm^3^, an adequate number of recent thymic emigrants (CD45RA), and a good response to the non‐live tetanus vaccine (antibody titer 3 weeks after the third dose of DTaP vaccine).^21^ However, it appears that most partial DiGeorge syndrome patients are vaccinated directly by primary care physician or public health clinic, without prior immunology workup and with a good tolerance to vaccination (summarized in Table S4).^6^, ^7^, ^9^, ^13^, ^16^ Nevertheless, it seems important to follow the listed criteria above as adverse severe reaction such as death, has been reported in a 13 months old child with MDS who was severely immunosuppressed (396 total T cells/mL; 320 CD4 T cells; 57 CD8 T cells).^22^ Further, another MDS patient with juvenile idiopathic arthritis and receiving weekly injections of the TNF inhibitor etanercept, has developed a skin rash with measles vaccine strain. His immunology workup showed a lymphocyte count of 1100 × 10^9^ cells/L, and normal lymphocyte subsets.^8^

The literature shows that children with DiGeorge syndrome have an increased risk of developing immunological changes over time, such as a decrease in their T‐cell reserve or in their pool of naive T‐cells.^23^ This places them at high risk of infection. There is also evidence of decreased B‐cell maturation.^11^ For these reasons, an immune check‐up is recommended once a year during childhood for these children depending on their immunological abnormalities.^24^

Some patients may respond less well to vaccination,^8^, ^11^, ^15^ or even lose their vaccine antibody levels over time, and require additional doses of vaccine.^10^ A study showed that out of 27 patients with a MDS, vaccinated against pneumococcus, 11 (41%) of them had 4 weeks later, an anti‐pneumococcal antibody response that was either absent (results below laboratory seropositive threshold) or poor (seropositive threshold reached but antibody level increased less than 4 times post‐vaccination).^15^

In healthy children, after receiving one dose of the MMR (measles, mumps, rubella) vaccine, approximately 93% of children develop immunity to measles, 78% to mumps, and 97% to rubella. A second dose increases immunity for measles to about 97% and ensures almost universal immunity to mumps and rubella among vaccinated individuals.^25^

Immunity against chickenpox is considered following an exposure to wild type varicella or following 2 doses of chickenpox vaccine.^26^ A study showed that out of 42 patients with a 22q11.2 microdeletion, with >500 CD4+ T cells/mm^3^, who had received 2 doses of MMR vaccines, 85% of the patients were still seroprotected against measles, mumps, and rubella 1 year after vaccination. However, after 2 years, only 35% met the criteria for seroprotection (>200 mLU/mL for measles, >20 RU/mL for mumps, >10 RU/mL for rubella).^10^ According to a study by Soshnick et al. (2021), around 7% of DiGeorge patients require immunoglobulin replacement therapy, such as patients with low IgG levels and those with low vaccine titers.^27^ These observations underline the fact that vaccine responses must be verified in these patients.

The goal of our study was to analyse the vaccine seroprotection in patients with a DiGeorge syndrome who had received standard vaccination according to their age and country (see Table S1). The study aimed to understand whether these patients are well protected against the different vaccine preventable diseases or whether they would need a closer follow‐up, such as regular vaccine serology assessment and additional doses of vaccines. In order to develop clear vaccine strategies for these children, we wanted to correlate the vaccine serology results to their immune parameters (lymphocyte subsets enumeration and dosage of immunoglobulins IgG, IgA, and IgM) as well as to their clinical data (age, gender, vaccination history, and infectious history).

## 2 METHODS

### 2.1 Study design

This was a prospective observational study in Geneva between February 2022 and April 2023, in children and young adults from 1 to 25 years old with a DiGeorge syndrome. Patients with a diagnosis of DiGeorge syndrome, followed in the unit of Pediatric Immunology, Vaccinology and Rheumatology of the Children's Hospital of the University Hospitals of Geneva, or followed in the foundation “Pôle autism”, specialized in the psychiatric care of children with MDS, were proposed to take part to our study. To be included in the study, the patients were required to have a vaccination record available, to be aged between 1 and 25 years old and have a genetic diagnosis of MDS. The study was approved by the institutional research ethics board (CCER 2021–02394) and all patients or their parents gave written consent.

### 2.2 Blood analyses

The following patient characteristics were recovered with a questionnaire: age, gender, ethnicity, number and date of last SARS‐CoV‐2 vaccination, history of chickenpox, history of autoimmune disease, immunosuppressive therapy, and infectious history.

A blood sample was then collected to perform the following analyses: (1) Concentrations of IgG, IgA et IgM; (2) Concentrations of CD3+, CD4+, CD8+ T cells, and CD19+ B cells; (3) Vaccine serology for diphtheria, tetanus, haemophilus influenzae type b (Hib) (for children younger than 5 years), measles, varicella, hepatitis A, hepatitis B, SARS CoV‐2 (Anti‐S Ig, Anti‐N Ig), and pneumococcus (serotype 4, 6B, 9V, 14, 18C, 19F, 23F). It should be noted that the tested pneumococcal serotypes are all included in the 13‐valent pneumococcal conjugate vaccine (PCV13), that was in used during the study.

The concentration of immunoglobulins IgG, IgA, and IgM was assessed in the laboratory of immunology and allergology of the University Hospital of Geneva. Lymphocytes subsets enumerations were performed at the laboratory of Flow Cytometry of the University Hospital of Geneva. Absolute cells count was assessed using a single plateform technique with counting beads. Briefly, 50 μL of whole blood was added to dried reagent Duraclone tubes containing the following anti‐human monoclonal antibodies: CD45‐KRO, CD3‐APC‐A750, CD8‐PB, CD4‐PC7, CD19‐APC‐A700, CD16‐ECD, CD56‐ECD, and HLA‐DR‐PE (custom made Duraclone tubes including counting beads from Beckman Coulter). Tubes were incubated for 15 min at room temperature following by a lyse‐no‐wash procedure (Versalyse, Beckman Coulter). Acquisitions of samples were directly performed on Navios cytometer (Beckman Coulter), and a gating on CD45‐SSC was used to analyse lymphocyte subsets.^28^ As for lymphocytes, each patient was classified as having either suboptimal (<N) or normal/high lymphocyte counts (≥N). The cut‐offs used for each lymphocyte are resumed in Table S2.

Vaccine serology tests were performed at the laboratory of Vaccinology of the University Hospital of Geneva. Serology tests were done using the Euroimmune enzyme‐linked immunosorbent assay (ELISA), except for pneumococcal serology which were done by Multiplex.^29^ Vaccine seroprotection was defined when the specific antibody (IgG) level was above the vaccinology laboratory's protection threshold, as described in Table S3.

Regarding pneumococcal seroprotection, in accordance with the usual practice of the University Hospital of Geneva, patients were considered protected if they had 4 or more serotypes with an IgG level ⩾0.5 mg/L out of the seven serotypes tested.

### 2.3 Vaccination guidelines

Patients were considered fully vaccinated for diphtheria, tetanus, *Haemophilus influenzae* type b, measles, varicella, pneumococcus, and hepatitis B if they had completed all the vaccinations recommended for their age according to the Swiss, Belgian, and French vaccination plan (see Table S1).^17^, ^18^, ^30^ The French and the Belgium recommendations for pneumococcal vaccination suggest that patients with primary immunodeficiency and more specifically hereditary primary immunodeficiency in French recommendations, such as patients with a DiGeorge syndrome, should receive the three doses of vaccine recommended for the general population before the age of 12 months, with an additional dose administered at 3 months old in France and as soon as the diagnosis is made for children older than 2 years old according to the Belgium vaccination plan.^17^, ^18^

Since vaccination against hepatitis A is not part of the basic vaccination, patients were considered fully vaccinated if they had received at least two doses, although this vaccine is only recommended in case of travel or for patients with hepatic diseases.^31^ Finally, in Switzerland, SARS‐CoV‐2 vaccine is recommended in case of non‐severe immunodeficiency, such as DiGeorge syndrome for children aged ≥5 years since December 2021.^32^ However, the recommendations that were in place at the beginning of the study have since been modified. The recommended doses have been changed from 2 doses to 3 doses in 2023.^33^ In our study, we considered patients who had received at least 2 doses of SARS‐CoV‐2 vaccine as fully vaccinated.

### 2.4 Statistical analysis

All analyses were performed using Stata version 17 (2021; StataCorp, College Station, TX). All associated graphs were produced using GraphPad Prism version 10.0.2 for Windows (GraphPad Software, San Diego, CA; www.graphpad.com). Continuous data were presented when appropriate as median and interquartile ranges (IQRs) (for age) or geometric mean concentrations (GMC) with the 95% CI for vaccine serology. Categorical data were presented as frequencies and percentages.

To analyse the temporal pattern of vaccine seroprotection, we performed Kaplan–Meier survival analyses for each vaccine type. The endpoint was defined as loss of seroprotection based on the laboratory thresholds (Table S3). Time was calculated from the last vaccine dose to either loss of seroprotection or last follow‐up (censoring). The number of subjects at risk was reported at 5‐year intervals.

## 3 RESULTS

### 3.1 Patient characteristics, infectious history and vaccine history

Patients' clinical data are summarized in Table 1. A total of 41 patients fulfilled our inclusion criteria and were enrolled in the study. The median patient age was 13 years old with an age range of 1–25 years. The vast majority of patients was Caucasian (93%). Thirty‐two patients (78%) had repeated infections in childhood up to 9.5 years on average (such as ear infections, bronchitis, pneumonia, or gastroenteritis) and 23 (56%) patients had a past history of varicella, among which very few (1%) had presented a severe varicella. No patients were taking immunosuppressive therapy at the time of testing.

**TABLE 1:** Characteristics of the study population.

Number of patients	41
Sex	
Male, *n* (%)	18 (44)
Female, *n* (%)	23 (56)
Age in years, median, [IQR]	13 [8–19.5]
Ethnic group	
Caucasian, *n* (%)	38 (93)
African, *n* (%)	2 (5)
Hispanic, *n* (%)	0
Asian, *n* (%)	2 (5)
Repeated infection in childhood, *n* (%)	32 (78)
Age at resolution, years, median	9
History of COVID‐19, *n* (%)	26 (63)
Severe infection, *n* (%)	0
History of chickenpox, *n* (%)	23 (56)
Severe infection, *n* (%)	4 (1)
Immunosuppressive treatment, *n* (%)	0
Vaccination guidelines followed^a^	
Diphtheria, *n* (%)	35 (85)
Tetanus, *n* (%)	35 (85)
Pneumococcus, *n* (%)	29 (71)
Haemophilus influenzae type b, *n* (%)	32 (78)
Measles, *n* (%)	36 (88)
Varicella, *n* (%)	27 (66)
Hepatitis A, *n* (%)	9 (23)
Hepatitis B, *n* (%)	21 (51)
SARS CoV‐2, *n* (%)	18 (44)

^a^ According to Swiss, Belgium, and French vaccination recommendations 2023 (resumed in Table 1).

Most patients were well vaccinated according to the Swiss, Belgian and French vaccination plan (see Table S1). Considering their age, 35 (85%) patients were fully vaccinated against diphtheria and tetanus, 36 (88%) against measles, 32 (78%) against *Haemophilus influenzae* type b, and 29 (71%) against pneumococcus with the vast majority receiving the PCV13 vaccine. Half were fully vaccinated against hepatitis B (51%), two‐third against varicella (66%) (meaning that they had either presented the varicella infection or had 2 doses varicella vaccine) and less than a quarter (23%) against hepatitis A. In addition, 44% had received at least 2 doses of SARS CoV‐2 vaccine, 63% had contracted COVID‐19 once or more, but none of them required hospitalization. Finally, it is important to note that most participants older than 5 years in our cohort had not received a 4th dose of pneumococcal vaccine, as recommended by the French and Belgium vaccination plans.^17^, ^18^

### 3.2 Vaccine seroprotection according to age

**FIGURE 1:**
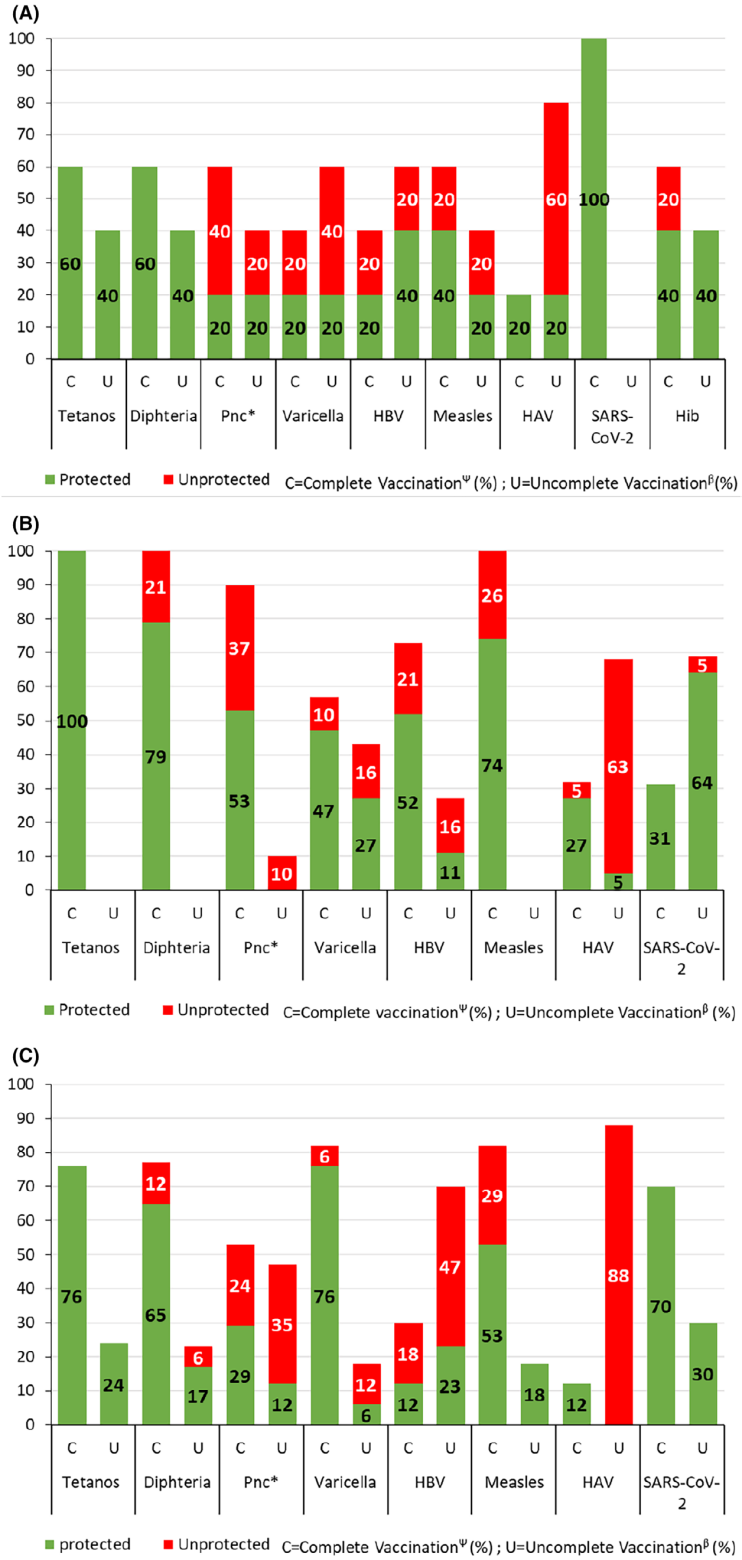
Percentage of patients seroprotected in green or unprotected in red for the various vaccine antigens in the group that received a complete vaccination (C) for their age, compared with the group that received an uncomplete vaccination^β^ (U), separated by age category, presenting data first for 1–5 (A), then 6–15 (B), and finally 16–25 years old (C). *Criteria for pneumococcus: Patients who had 4 or more serotypes that showed an IgG level ⩾0.5 mg/L out of the seven serotypes tested. ΨAccording to Swiss, Belgium and French vaccination recommendations 2023.^30^

**FIGURE 2:**
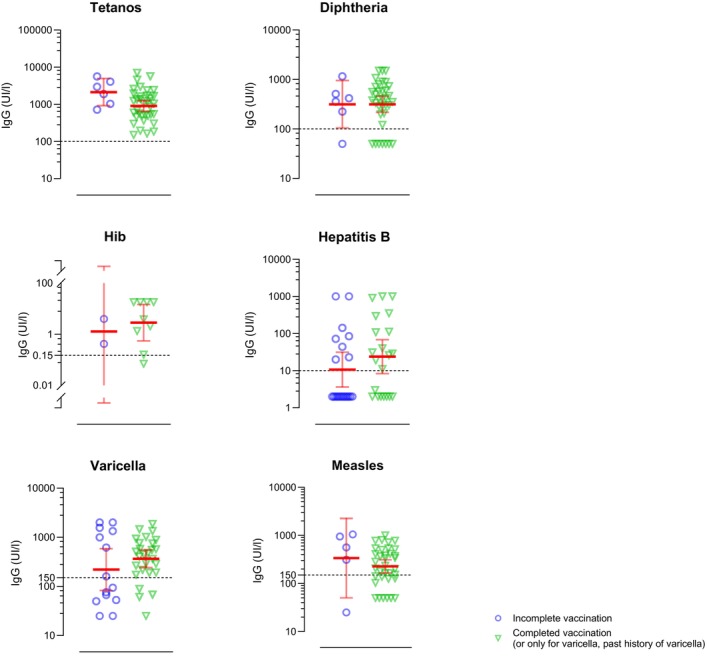
IgG levels against tetanos, diphtheria, haemophilus influenzae type b (Hib), hepatitis B, varicella and measles with geometric mean concentration and 95% confidence interval, presenting data first for the incompletely vaccinated group and then for the fully vaccinated group. The dashed line represent the seroprotection threshold.

**FIGURE 3:**
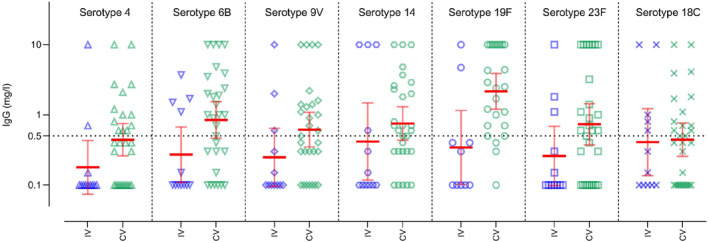
IgG levels with geometric mean concentrations and [95% confidence interval] for the different serotypes of pneumococcus, presenting data first for the incompletely vaccinated (IV) group in blue and then for the fully vaccinated group (CV) in green. The dashed line represent the seroprotection threshold.

All patients had vaccine serology available. The vaccine seroprotection for the various diseases was classified by age (category: 1–5 years, 6–15 years, and 16–25 years) and by vaccination status (see Figures 1, 2, 3).

Regarding tetanus, all patients were seroprotected regardless of their age and vaccination status. Concerning diphtheria, 34 patients (83%) had protective antibody level. All children under 5 were seroprotected.

Vaccine serology for *Haemophilus influenzae* type b was performed in children under 5 years old only. For these children, one in five was not seroprotected despite full vaccination.

For varicella, more than a quarter (27%) of patients were not seroprotected. Among them, 55% had neither a history of chickenpox nor had they been fully vaccinated,18% had received an incomplete vaccination, 18% had chickenpox, and finally 1 child was not seroprotected despite having been fully vaccinated. In the group younger than 5 years, 60% were not seroprotected, but two‐thirds of the unprotected had neither a history of chickenpox nor had they been fully vaccinated. In the 6–15 age group, 1 in 4 were not seroprotected. However, it should be noted that 1/3 of these non‐seroprotected children had received a full vaccination or had been exposed to varicella. Finally, for young adults aged 16 and over, more than 80% were seroprotected (see Figure 1).

One third of our patients were not seroprotected against measles despite complete vaccination in most of them (Figure 2). Among children under 5, 40% were not seroprotected although half had been vaccinated completely. All children aged 6–15 had received a full vaccination for their age; however, 1 in 4 were not seroprotected. Similarly, for children aged 16 and over, the third who were not seroprotected were all vaccinated.

**FIGURE 4:**
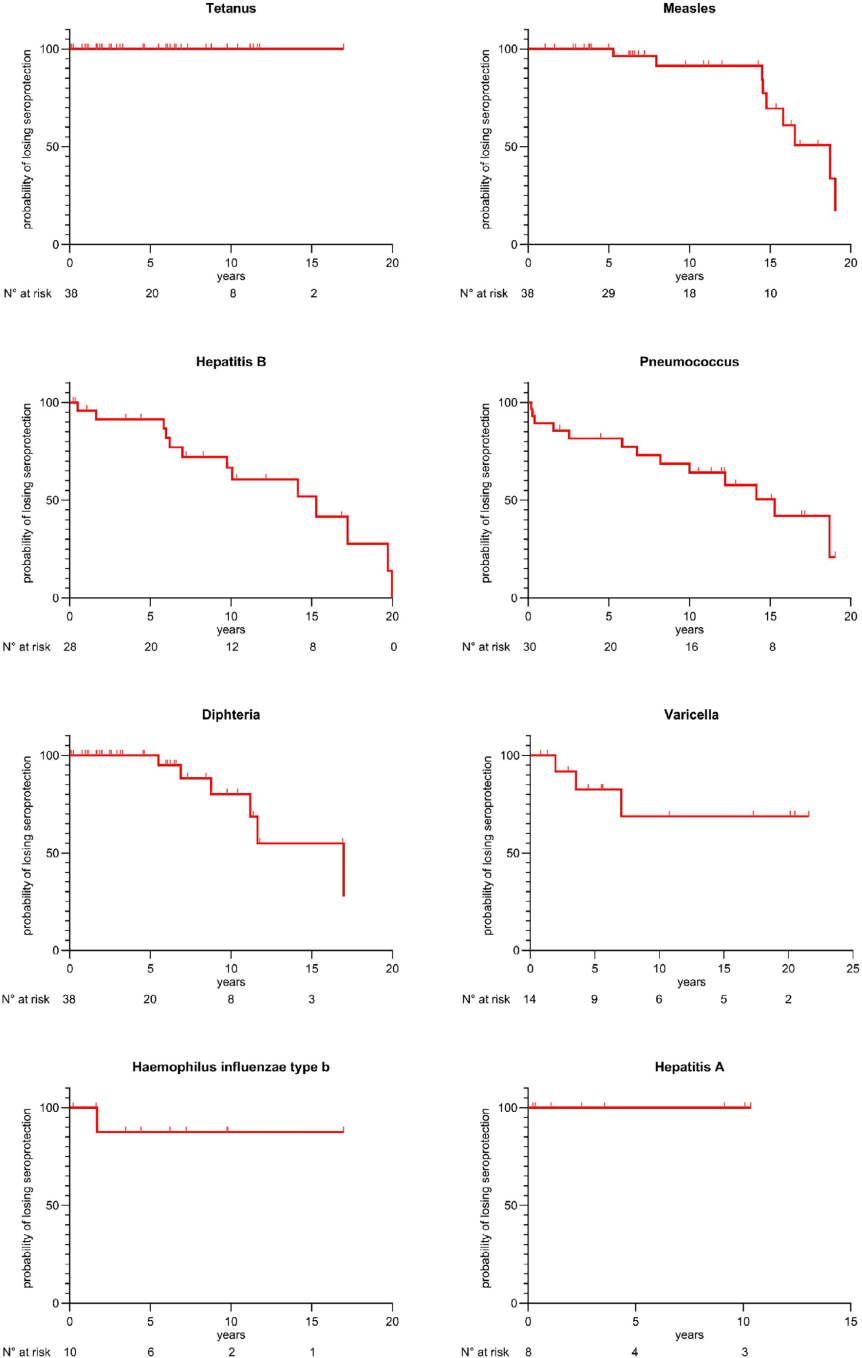
Kaplan–Meier curves showing the probability of maintaining seroprotection over time for different vaccine antigens in MDS patients. The number of patients at risk is shown below each graph.

Moreover, half of the patients (49%) in the study had insufficient protection against hepatitis B. Among individuals with a complete vaccination, 13/21 (62%) were seroprotected, while 8 (38%) remained unprotected. In the under 16 years, around 40% were not seroprotected, while half of them had received a full vaccination for their age. Among young adults aged 16 and over, 2/3 were not seroprotected, but the majority had not received a full vaccination.

In addition, very few patients were protected against hepatitis A (*n* = 10; 24%); however, almost all of these children had not received a full vaccination. It is important to note that complete vaccination against hepatitis A resulted in seroprotection in all patients (8/8, 100%).

Concerning pneumococcus, half of the patients were not seroprotected against pneumococcus (*n* = 22; 54%). For children under 5 years of age, 3 out of 5 (60%) were not seroprotected, although 2/3 of these children had received a full vaccination. Among patients aged 6–15 years, 1 in 2 (47%) were not seroprotected against pneumococcus, although 80% of these children had received at least 3 doses of pneumococcal vaccine. For young adults aged 16 and over, 3 out of 5 were not seroprotected, although 40% of them had received at least 3 doses of pneumococcal vaccine in infancy. For pneumococcus, among individuals with a complete vaccination (defined as at least 3 doses of pneumococcal vaccine), 15/29 (52%) were seroprotected, while 14 (48%) remained unprotected.

Finally, regarding SARS‐CoV‐2, almost all patients were seroprotected (*n* = 40; 98%), with 40 (98%) having anti‐S antibodies and 36 (88%) having anti‐N antibodies.

The geometric mean concentration with the 95% confidence interval of vaccine antibody levels for the different vaccine antigens tested are described on Figures S1 and S2.

### 3.3 Immunological parameters

The immunological data, including the serum immunoglobulin levels (IgG, IgA, and IgM), the total lymphocyte count, and the subsets of T and B lymphocytes counts, are described in Table 2. The results showed that most patients with MDS exhibited normal levels of immunoglobulins according to age (see Table S2), with 40/41 (98%) for IgG, 35/41 (86%) for IgA, and 27/41 (66%) for IgM. These findings indicate a normal quantitative humoral immune capacity in most patients. Regarding the lymphocyte subsets, the median total lymphocyte count was 2280 cells/mm3, with a range of 1688–2675 cells/mm^3^ (IQR). Most patients (36/41, 88%) had total lymphocyte counts above the established threshold according to age (described in Table S2), indicating normal lymphocyte levels. Analysis of T lymphocyte subsets revealed that 34/41 patients (84%) had normal CD3+ T lymphocyte counts. Furthermore, 35/41 patients (85%) exhibited normal CD4+ T lymphocyte counts, and 37/41 patients (90%) had normal CD8+ T lymphocyte counts. These results showed that most patients had normal T cells subsets. Lastly, 39/41 patients (96%) had normal B lymphocyte counts, indicating the absence of B cell lymphopenia.

**TABLE 2:** Number and percentage of patients with age‐appropriate normal levels of Immunoglobulins and Lymphocytes.

	Patients, *n* (%)
Immunoglobulins	
IgG	40 (98)
IgA	35 (86)
IgM	27 (76)
Lymphocytes	
Total lymphocytes	36 (88)
T lymphocytes CD3+	34 (83)
T lymphocytes CD4+	35 (85)
T lymphocytes CD8+	37 (90)
B lymphocytes CD19+	39 (96)

### 3.4 Vaccine seroprotection persistence over time

Given that the time elapsed since the last vaccination is crucial for understanding immunity waning patterns in this population, we analysed the temporal relationship between vaccination and seroprotection, through Kaplan–Meier survival analyses for each vaccine antigen (see Figure 4). This analysis revealed distinct temporal patterns across different vaccines. Tetanus vaccination showed sustained seroprotection rates throughout the follow‐up period. For measles, the probability of maintaining seroprotection decreased markedly after 12 years post‐vaccination. Hepatitis B protection showed a gradual decrease over time, with a notable decline observed after 10 years post‐vaccination. The pneumococcal vaccine protection analysis revealed a decline within the first 5 years after vaccination. Diphtheria protection remained above 90% for the first 10 years, followed by a decrease in the probability of maintaining seroprotection. The analyses for varicella, Haemophilus influenzae type b, and hepatitis A vaccines, while included, require cautious interpretation due to the limited sample size.

## 4 DISCUSSION

### 4.1 Infectious history

Our results showed that patients with a DiGeorge syndrome present recurrent otitis and upper respiratory infections especially during the first decade of their life. Similarly, previous studies have shown that patients with a MDS are more likely to have recurrent infections, including vaccine‐preventable infections.^3^, ^34^ A large study carried out in Europe showed that only 7% of patients with a DiGeorge syndrome had recurrent infections.^35^ However, another study reported that recurrent sinusitis, otitis, and lower airway infections affected 25%–30% of DiGeorge patients over 9 years of age and into adulthood.^36^ These results underline the importance of ongoing immunological surveillance at least during the first decade of life and targeted interventions to enhance vaccine protection in this population.^24^

### 4.2 Immune capacity

Previous studies have shown that patients with a MDS could develop immunological changes over time, with a decrease in the T‐cell repertoire and a decrease in the pool of naive T‐cells leading to an increased risk of infections.^23^, ^37^ Further, it has been reported previously that these patients have a decrease in B cell maturation.^11^ However, in our study, more than 80% of the patients had normal T cells subsets and nearly all our patients had normal B lymphocyte counts. Concerning the levels of immunoglobulins, a large study conducted by the European Society for Immunodeficiency and the US Immunodeficiency Network, involving 1023 patients diagnosed with a DiGeorge syndrome, revealed that only a small proportion of children older than 3 years had a decreased immunoglobulin levels (6.2% for IgG, 1.3% for IgA, and 23% for IgM),^38^ similarly to our study.

### 4.3 Vaccination status

Data on vaccination status of patients with a DiGeorge syndrome are lacking except for live vaccines. A suboptimal vaccine coverage has been reported in this population, especially for live vaccines.^7^, ^9^, ^14^ In contrast, it is reassuring to report that in our study, most patients were up to date with their vaccines, included MMR; however, half had not been sufficiently vaccinated against hepatitis B or SARS‐CoV‐2. This lack of vaccination against SARS‐CoV‐2 can probably be explained by the fact that around 60% of the patients had already been exposed at least once to the virus and therefore did not need to have the second recommended dose of COVID‐19 vaccine. However, it is concerning to note that children with a MDS are not fully up to date with their hepatitis B vaccination. Similarly, despite not having chickenpox as children, one‐third of the patients did not receive a complete varicella vaccination. In fact, among non‐seroprotected children, more than half had no prior vaccination or past infection, although the median age of the patients were 13 years. It should be noted that since 2023, the Swiss vaccination plan has recommended 2 doses of varicella vaccine at 9 and 12 months for all children, which is not the case currently in France and Belgium.^39^ Pediatricians should be encouraged to follow the vaccination recommendations in force in their country and recommend varicella vaccination for patients with DiGeorge syndrome in the absence of evidence of past infection and if they have sufficient T cell count. In Switzerland and in France, hepatitis A vaccination is a complementary vaccine, that is only recommended in case of expositional risk factors or past hepatic disease,^18^, ^40^ which explains why only one quarter of our patients were vaccinated against hepatitis A. However, it is important to note that in Belgium, it is recommended that immunocompromised people are vaccinated against hepatitis A.^41^

Finally, it was alarming to see that around one third of the patients had not received 3 doses of pneumococcal vaccine recommended for the general population, and the majority had not received the 4th additional dose recommended for the MDS patients. An additional dose of pneumococcal vaccine should be given to all MDS patients.

### 4.4 Vaccine seroprotection

Firstly, regarding live vaccines, a study has analysed the immune response of MDS patients after measles vaccination and shown that despite repeated vaccination, long‐term protective measles antibody levels appear far lower than those in the general population at the same age.^10^ High measles vaccination coverage was observed in the 22q11.2 population in our study, but only one‐third of fully vaccinated patients had adequate measles seroprotection. These results suggest a poor response to measles vaccine or an early loss of vaccine antibodies. Given the high mortality (1:3000 cases) and morbidity of measles, it is crucial that all children stay protected against this terrible infection.^42^ We propose that patients with DiGeorge syndrome should be periodically assessed for measles antibody levels, especially during adolescence, according to our Kaplan–Meier survival analysis.

No study has described the prevalence of seroprotection against varicella in this population. However, in our study, one seventh were not seroprotected despite complete vaccination or past infection. Nevertheless, it is reassuring to report that very few children (1%) developed severe chickenpox, and the vast majority of children over 16 years of age were well seroprotected. These results show that for patients with MDS, the infectious or vaccination history of varicella might be unreliable. When possible, vaccine serology should be carried out regularly (maybe every 3–5 years) in order to improve vaccine immunity.

Concerning the other vaccine antigens, in our patient cohort, almost all the children fully vaccinated were seroprotected against diphtheria and tetanus. Similarly, two studies in patients with MDS reported a good antibody response to tetanus and diphtheria.^10^, ^16^ For *Haemophilus influenzae* type b, most individuals with complete vaccination were seroprotected. These results suggest that three doses of vaccine before 12 months of age are probably sufficient to protect this population during the first 5 years of life.

Currently, data on SARS‐CoV‐2 infections in DiGeorge patients are scarce and limited to one case report and one study on adult patients, which did not observe a higher risk of severe infections or hospitalization in this population.^43^, ^44^ Moreover, data on immunological response to SARS‐CoV‐2 vaccine are lacking. A study in adults has shown that 22q11.2 patients can induce a robust specific IgG response after vaccination with the mRNA‐SARS‐CoV‐2 vaccine.^44^ Our study showed that almost all the patients were seroprotected against SARS‐CoV‐2. It is reassuring to see that many had been exposed to the SARS‐CoV‐2 but none had presented severe symptoms.

Concerning hepatitis A, only one quarter of the patients were seroprotected because this vaccine is not routinely recommended for healthy people and patients with DiGeorge.

In our study, we found that half of the patients were not seroprotected against hepatitis B, partly due to the fact that half of the patients were not sufficiently vaccinated for their age according to the Swiss, Belgium, and French national immunization program. Particularly, most young adults aged 16 and over were not seroprotected, mainly due to a lack of complete vaccination. Hence, pediatricians and family physicians are advised to pay particular attention to the vaccination of young adults with MDS. However, we observed that despite full hepatitis B vaccination, more than a third of the MDS patients were no longer protected. Similarly, a study carried out in Taiwan on a pediatric population of 87 DiGeorge patients showed that 46% had a lack of hepatitis B antibody.^37^ In view of these results, we propose that patients with DiGeorge syndrome should be periodically assessed for hepatitis B antibody levels regardless of vaccination status and reimmunized accordingly. Indeed, like other immunocompromised patients, they should probably have consistently antibodies anti‐HBV above 10 IU/L.^31^

Finally, half of our patients were not seroprotected against pneumococcus despite a complete vaccination. In comparison, a previous study has shown that patients with a MDS had an abnormal response to pneumococcal vaccine.^15^ Given the high frequency of mild‐to‐moderate immunodeficiency in this population, their vulnerability to sinopulmonary infections, and their underlying anatomical dysfunction suggest that more investigations should be performed to better understand pneumococcal vaccine response in this population and at which frequency, additional pneumococcal vaccine doses should be administered.^3^, ^45^

Our study is limited by the fact that we do not have a control group of healthy children of similar age and demographics for comparison. Another limitation is the fact that we only assessed vaccine antibody concentrations, but we did not assess the functional quality of the immune response (e.g., neutralizing capacity of the antibody or the memory T/B cell responses). It would be very important to develop functional assays to assess the quality of the antibody responses and the long‐term induction of immunity through the measurement of memory B and T cells for immunocompromised patients, such as MDS patients.

In conclusion, while standard vaccination regimens have shown effectiveness across different age groups in providing substantial seroprotection against tetanus, diphtheria, and SARS‐CoV‐2, suboptimal seroprotection against measles, pneumococcal, and hepatitis B have been observed, despite good vaccine coverage. These results suggest a premature waning of vaccine‐induced antibodies. Furthermore, reliance solely on the vaccine or infection history (especially for chickenpox) appears unreliable for assessing vaccine seroprotection. Therefore, we advocate for a proactive approach, including regular assessment of measles, hepatitis B, varicella, and pneumococcal antibody levels, irrespective of vaccination status, and re‐immunization according to the results. Further investigation is warranted to explore optimal vaccine strategies aimed at enhancing vaccine immunity in this population.

## AUTHOR CONTRIBUTIONS

**Leila Farpour:** Conceptualization; investigation; writing – original draft. **Renato Gualtieri:** Investigation; writing – review and editing; formal analysis. **Tereza Kotalova:** Investigation; writing – review and editing. **Barbara Lemaître:** Investigation; writing – review and editing. **Julie Ducreux:** Investigation; writing – review and editing. **Isabelle Arm‐Vernez:** Investigation; writing – review and editing. **Stephan Eliez:** Conceptualization; investigation; writing – review and editing. **Geraldine Blanchard‐Rohner:** Conceptualization; investigation; funding acquisition; validation; writing – review and editing; formal analysis; supervision; resources.

## CONFLICT OF INTEREST STATEMENT

The authors declare that they have no conflicts of interest to disclose as described by Pediatric Allergy and Immunology.

## Supporting information


Figure S1.

Table S1.
