# Supplementary material for: Vaccine immunity in patients with 22q11.2 microdeletion syndrome
Source: Pediatr Allergy Immunol. 2025 Feb 10;36(2):e70043. doi: 10.1111/pai.70043 (PMC12813736; doi:10.1111/pai.70043)
Supplement: Supplementary file 1 — Figure S1. [file PAI-36-e70043-s001.docx]

**Supplementary Figure 1**: Geometric concentrations (GMCs) and [95% confidence interval] of IgG concentrations for the various vaccine antigens, presenting data first for the incompletely vaccinated group and then for the fully vaccinated group.

|  | Incomplete vaccination | Complete vaccination |
| --- | --- | --- |
| Tetanus | 2136 [924.4 to 4934] | 908.1 [644.2 to 1280] |
| Diphtheria | 316.0 [104.9 to 951.8] | 315.4 [216.9 to 458.6] |
| Haemophilus influenza b | 1.3 [8.1x10^-7^ to 2.0x10^6^] | 2.8 [0.5 to 14.6] |
| Hepatitis B | 10.7 [3.6 to 31.4] | 23.9 [8.3 to 68.7] |
| Varicella | 220.0 [83.0 to 586.1] | 364.2 [241.7 to 548.7] |
| Measles | 337.4 [50.3 to 2263] | 226.8 [165.4 to 311.1] |

**Supplementary Figure 2:** Geometric mean concentrations (GMCs) and [95% confidence interval] of vaccine serology for the different serotypes of pneumococcus, presenting data first for the incompletely vaccinated (IV) group and then for the fully vaccinated group (CV).

|  | Incomplete vaccination (IV) | Complete vaccination (CV) |
| --- | --- | --- |
| Serotype 14 | 0.4 [0.1 to 1.5] | 0.7 [0.4 to 1.3] |
| Serotype 19F | 0.3 [0.1 to 1.2] | 2.1 [1.2 to 3.9] |
| Serotype 23F | 0.3 [0.1 to 0.7] | 0.7 [0.4 to 1.4] |
| Serotype 4 | 0.2 [0.1 to 0.4] | 0.4 [0.3 to 0.7] |
| Serotype 6B | 0.3 [0.1 to 0.7] | 0.8 [0.5 to 1.5] |
| Serotype 9V | 0.2 [0.1 to 0.6] | 0.6 [0.3 to 1.0] |
| Serotype 18C | 0.4 [0.1 to 1.2] | 0.4 [0.3 to 0.8] |
