# Supplementary material for: Vaccine immunity in patients with 22q11.2 microdeletion syndrome
Source: Pediatr Allergy Immunol. 2025 Feb 10;36(2):e70043. doi: 10.1111/pai.70043 (PMC12813736; doi:10.1111/pai.70043)
Supplement: Supplementary file 2 — Table S1. [file PAI-36-e70043-s002.docx]

**Appendix**

**Supplementary table 1**: Recommended vaccinations according to the Swiss, Belgian and French vaccination plans 2023^17,18,38, 40, 45, 46^

| **Vaccine** | **Recommendations** | **Comments** |
| --- | --- | --- |
| Diphtheria-tetanus-acellular pertussis vaccine | 3 doses of combined vaccine during first year of life  +  Switzerland: one dose between 4-7 years old  Belgium and France: one dose at 5-6 years old  +  one dose during adolescence and then one dose at 25, 45 and >65 years old | In Belgium, a dose is also recommended at 15 months in addition to basic vaccination for all children.  Recommendations for DiGeorge children are identical to those for the general population |
| Hib vaccine | 3 doses during first year of life. | In Belgium, a dose is also recommended at 15 months in addition to basic vaccination for all children.  Recommendations for DiGeorge children are identical to those for the general population. |
| Inactivated polio vaccine | 3 doses during first year of life and one dose between 4-7 years old. | In France, an additional dose is given during adolescence for all children. |
| HBV vaccine | 3 doses of combined vaccine during first year of life | In all three countries, vaccination is proposed to adolescents if not received during infancy.  In Belgium, a dose is also recommended at 15 months in addition to basic vaccination for all children. |
| Pneumococcal conjugate vaccine | 3 doses of PCV13 during first year of life | In France, for children with hereditary primary immunodeficiency, a fourth dose of pneumococcal conjugate vaccine is recommended at 3 months of age and a booster vaccination every 5 years with the 23-valent plain polysaccharide vaccine.  In Belgium, for children with primary immunodeficiency such as DiGeorge syndrome, an additional dose of the 23-valent plain polysaccharide vaccine is given at diagnosis if older than 2 years, and a booster vaccination with the 23-valent plain polysaccharide vaccine is recommended every 5 years for children >16 years old at increased risk of pneumococcal infection. |
| Live attenuated MMR vaccine | 2 doses are recommended before adolescence. | - Switzerland: at 9 and 12 months - France: at 12 and between 16-18 months - Belgium: at 12 months and at 7-8 years old |
| Live attenuated varicella vaccine | 2 doses are recommended during adolescence if no history of varicella during childhood |  |

**Supplementary table 2**:

| **Cut-off used for absolute size of lymphocyte subpopulations and immunoglobulins concentration^61^** | | | | | | | |
| --- | --- | --- | --- | --- | --- | --- | --- |
|  |  | **Age groups** | | | | | |
| **Lymphocyte subpopulations *** | | **9-15 mo** | **15-24 mo** | **2-5 yr** | **5-10 yr** | **10-16 yr** | **Adults** |
| Total lymphocytes | | ≥ 2600 | ≥ 2700 | ≥1700 | ≥ 1100 | ≥ 1000 | ≥ 1000 |
| T lymphocytes CD3 | | ≥ 1600 | ≥ 1400 | ≥ 900 | ≥ 700 | ≥ 800 | ≥ 700 |
| T lymphocytes CD4 | | ≥ 1000 | ≥ 900 | ≥ 500 | ≥ 300 | ≥ 400 | ≥ 300 |
| T lymphocytes CD8 | | ≥ 400 | ≥ 400 | ≥ 300 | ≥ 300 | ≥ 200 | ≥ 200 |
| B lymphocytes CD19 | | ≥ 600 | ≥ 600 | ≥ 200 | ≥ 200 | ≥ 200 | ≥ 100 |
| **Immunoglobulins concentration** | |  |  |  |  |  |  |
| IgG (g/l) | | ≥ 3.3 | ≥ 3.3 | ≥ 4.8 | ≥ 5.5 | ≥ 6.5 | ≥ 6.5 |
| IgA (g/l) | | ≥ 0.2 | ≥ 0.2 | ≥ 0.3 | ≥ 0.4 | ≥ 0.5 | ≥ 0.7 |
| IgM (g/l) | | ≥ 0.5 | ≥ 0.6 | ≥ 0.7 | ≥ 0.8 | ≥ 0.9 | ≥ 0.10 |
| *Absolute counts (cell/mm3) | |  |  |  |  |  |  |

**Supplementary table 3**:

| **Vaccine** | **Protection Threshold** |
| --- | --- |
| Diphtheria | ≥100 IU/L |
| Tetanus | ≥100 IU/L |
| Measles | ≥150 IU/L |
| Varicella | ≥150 IU/L |
| Hepatitis A | Qualitative (Protected = Positive) |
| Hepatitis B | ≥10 IU/L |
| Haemophilus influenzae type b* | ≥0.15 mg/L |
| SARS CoV-2 | Anti-S >0.9 IU/mL and/or Anti-N >1.10 IU/mL |
| Pneumococcus | ≥0.5 mg/L in ≥4 of 7 tested serotypes |

*Measured only in patients <5 years of age

**Supplementary table 4:** previous vaccine studies in MDS patients

| **Citation, Date, Country, Study design** | **Vaccines studied** | **Study group, median age** | **Outcome** | **Main results** | **Comments** |
| --- | --- | --- | --- | --- | --- |
| **Mc Gregor et al. (2022), [6], Canada**  Retrospective study | Rotavirus | 42 children with MDS,  Median age at vaccination: 2 months | Adverse events | 22/39 (56%) received at least one dose of the rotavirus vaccine. No adverse events.  43% of patients with CD4+ >500 cells/µL did not receive the vaccine despite meeting safety criteria.  26/39 patients had immunology workup:  - 2/39 Lower CD4+ T cells  - 2/39 Lower CD8+ T cells | Only 10/26 (38%) underwent immune workup before receiving a live vaccine. |
| **Berkhout et al. (2020) [7], Australia**  Retrospective cohort study | MMR,  Varicella,  Pneumococcal vaccines | 134 children with MDS,  No median age mentionned | Live vaccines and pneumococcal vaccines coverage,    Adverse Events | 102/134 (82%) patients with MDS vaccinated with a LAV.   - MMR (102) - Varicella (82)   No significant adverse events, including in children with CD4 counts <0.5×10⁹/L (2/102).  Additional pneumococcal vaccination:  22/125 (18%) received a 4^th^ dose PCV-7 or 13  16/103 (16%) received a dose of PCV23 from 4 years of age. | Immunology work-up prior to live vaccines was inconsistent (25% for MMR, 33% for Varicella). |
| **Iroh Tam et al. (2015) [8], USA,** Retrospective study | Hib,  PCV-7 and PCV-13,  Diphtheria,  Tetanus | 12 children with MDS  Mean age at vaccine titer evaluation: 3 years and 10 months. | Vaccine response, Humoral immunity | All had protective responses to diphtheria and tetanus vaccines.  3/12 had non-protective titers to Hib vaccine  3/12 had protective titers to >50% of pneumococcal serotypes  11/12 had normal T, B, and NK cells  11/12 had decreased immunoglobulin levels | 1 child did not respond to any pneumococcal serotypes and to Hib vaccine, despite normal T, B, and NK cells and normal immunoglobulins. |
| **Hofstetter et al. (2014) [9], USA**  Multicenter retrospective cohort study | LAV :  MMR,  Varicella,  OPV,  Rotavirus  IPV | 194 subjects with MDS,  Aged between 0-31.5 years  Mean observation period: 8.3 years | Adverse Events | 90% received at least one live vaccine.   - MMR (141) - Varicella (98) - OPV (52)   No significant adverse events, including in children with CD4 counts <15% (3/141)  Lower CD4 associated with varicella AE (fever, skin rash), no MMR AE.  Half had lymphocyte screening:  CD4+ T-cells categories (% of total T cells) :   - 6%: <15% (severe immunodepression), - 35%: 15–24% (moderate immunodepression), - 80%: ≥25% (no immunodepression) | Clinical varicella in 11/59 non-immunized and was mild.  Early FISH confirmation was associated  with lower MMR and varicella  vaccination coverage and delayed immunization.  Lymphocyte screening before vaccination not systematically done. |
| **Al-Sukaiti et al. (2010) [10], Canada**  Retrospective observational study | MMR | 82 patients with MDS  Median age: 5.5 years | Adverse Events  Sustaining antibodies | Mild reactions (fever, skin rash) in 6/82 patients (7.3%).  No moderate or severe reactions observed.  Similar reaction rate to general population.  11/12 robust seroconversion to MMR 1 year after MMR vaccination.  Difficulty in sustaining long-term antibodies:   - 10/29 seroprotected 2 years after MMR vaccination | Most patients had adequate T-cell counts (>500 CD4 cells/mm³).  81/82 Normal CD8+ T-cells subset. |
| **Finocchi et al. (2006) [11], Italy,**  Observational study | Hib,  PCV23 | 13 children with MDS,  Median age : 3 years and 2 months | Vaccine responses, Humoral immunity | 2/13 had impaired response to Hib vaccine  3/13 had impaired responses to pneumococcal vaccine  13/13 patients had immunology workup:   - 43% showed decreased Igs - 7/13 Lower CD3+ and CD4+ T cells; - 2/13 Lower B cells; - Normal polyclonal lymphoproliferative assays. | 9/13 patients had recurrent infections.  2/13 required intravenous immunoglobulin (IVIG) therapy every 4 weeks  5/13 required daily prophylactic antibiotics. |
| **Azzari et al. (2005) [12], Italy**  Prospective study | MMR | 14 children with MDS  Mean age : 34 months | Adverse events,  Immunogenicity | No significant adverse events  Seroconversion rates:  - Measles 13/14 (93%),  - Rubella 13/14 (93%), comparable to healthy controls.  No decrease in CD4 count observed after vaccination. |  |
| **Moylett et al. (2004) [13], USA**  Retrospective  Based on questionnaire to parents and primary care physicians records | LAV:  MMR  Varicella  OPV  IPV | 53 Children with MDS  Median age at vaccination:  - 13 months MMR  - 2,4,6 m OPV  -12.5 m VZV | Adverse events | 25/53 (47%) patients with MDS vaccinated with a LAV.   - MMR (25) - Varicella (13) - OPV (6)   No significant adverse events  15/25 patients had immunology workup:   - Lower T cells subsets - Normal polyclonal lymphoproliferative assays but 3 patients had lower antigen specific responses. | Only 15/25 vaccinated knowing the diagnosis.  Only 3 patients were recommended LAV based on T cell count.  22 patients vaccinated by primary care physician or public health clinic.  VZV immunoglobulin administered post-exposure to 5/40 non-immunized children.  Clinical varicella in 4/40 non-immunized children and was mild. |
| **Perez et al. (2003) [314], USA,** Retrospective study | LAV:  MMR, Varicella | 59 children with MDS,  median age: 6 years | Adverse Events | 52/59 patients with MDS vaccinated with a LAV:   - MMR (52) - Varicella (32)   No significant adverse events  Similar reaction rate to general population. | All clinical varicella was mild.  None of the varicella vaccinated children developed wild-type disease. |
| **Gennery et al. (2002) [15], UK**  Retrospective observational study | Tetanus,  PCV | 32 patients with MDS, median age: 6 years | Vaccine response Recurrent infections | 11/20 poor antibody response to PCV *  3/32 poor antibody response to tetanus*  13/32 had decreased immunoglobulins | 26/32 had severe or recurrent infections (sinopulmonary system)  50% of patients with recurrent infections had decreased Immunoglobulin levels.  34% required daily prophylactic antibiotics to manage severe or recurrent infections. |
| **Junker et al. (1995), [16], Canada/USA,** Retrospective study | MMR,  Tda,  Poliovirus,  VZV, | 13 children with MDS,  aged between 6-18 years at the time of the survey | Vaccine-induced immunity,  Humoral immunity | Good antibody responses to:   - Diphtheria (13/13), - Tetanus (13/13), - Poliovirus types 1 (5/9), type 2 (9/9) and type 3 (8/9), - MMR (11/13), - VZV (5/5),   Antibody avidity for rubella, VZV, and CMV was comparable to controls.  13/13 patients had immunology workup:   - 5/13 Lower B -cells subset; - 2/13 Lower IgG; - 2/13 Higher IgA. | All children vaccinated by primary care physician or public health clinic.  Only 6/13 developed antibodies to all seven vaccine antigens tested  No correlation between antibody responses and numbers of T  CD4 + cells. |

Tda = tetanus, diphtheria and acellular pertussis; MMR = measles, mumps and rubeola; PCV = pneumococcal conjugated vaccine; Hib = *Haemophilus influenzae* type B; IPV = inactivated poliovirus; MDS = microdeletion 22q11.2 syndrome ; OPV = oral poliovirus ; LAV : live attenuated vaccine ; PCV23= 23-valent pneumococcal polysaccharide vaccine; PCV-7 =7-valent pneumococcal polysaccharide vaccine, PCV-13= 13-valent pneumococcal polysaccharide vaccine; *= less then 2 standard deviations below the age related geometric mean.
